# Improving the hospital or immunizing its organization? Patient and public involvement at the service of quality

**DOI:** 10.3389/fsoc.2024.1444955

**Published:** 2024-11-11

**Authors:** Bernard Voz, Benoît Pétré, Jean-François Orianne

**Affiliations:** ^1^Department of Public Health Sciences, Faculty of Medicine, University of Liege, Liège, Belgium; ^2^Institute of Research in Social Sciences, Faculty of Social Sciences, University of Liege, Liège, Belgium

**Keywords:** patient and family advisory council, hospital, patient involvement, quality, systems theory, differentiation, organizational system, immune function

## Abstract

**Introduction:**

There is a pressing need for the hospitals to improve their quality and become more patient-centered. Over the last decade, several approaches were implemented to meet this demand, such as hospital accreditation or patient surveys. Many studies have addressed the patient involvement systems from the viewpoint of the factors that drive them or the achieved performance. In this study, we examined the patient involvement from the viewpoint of its function and operation rather than its performance. Following Luhmann, we reconsidered quality to be related to the absorption of uncertainty rather than improvement or innovation. The adaptation of an organization to involve patient participation can be regarded as contributing to the immune function of the organizational system.

**Methods:**

Three case studies addressing patient and family advisory councils in general hospitals were conducted in Belgium. Qualitative empirical material is retrieved from observation, documentation, and interviews.

**Results:**

Our findings suggest that the immune function of the hospital organization operates in four main phases. First, we assess how the communicative process indicates the relevant difference that needs to be addressed. Role differentiation occurs through the depoliticization and depersonalization of criticism. Second, given the impossible realization of first-order observation of the environment, our material shows how second-order observation is organized through a dual representation. Third, we unveil how the environmental representation requires a specific organizational socialization to overcome the representation paradox. Finally, we analyze how the whole described process must fulfil the preparation of a repertoire of responses to the irritations of its environment.

**Discussion:**

The analysis revealed that patient and family advisory councils complete a crucial immune function for organizations, far beyond the simple discussion of the “nuts and bolts” of organizational structure. These findings permit to discuss implications of the notions of participation and quality regarding to identity work of stakeholders, open organization, and change management.

## 1 Introduction

After achieving initial developments in the industry and commerce fields (Karpik, 2010), the quality concept has spread to other sectors. In the healthcare field, the embracement of quality has gradually occurred since the first publications by Donabedian (1966) to the landmark reports by the US Institute of Medicine (Institute of Medicine US, 2000, 2001) which marked the definitive recognition of quality as a genuine paradigm for collective action (Setbon, 2000). The links between the pursuit of quality and the universal healthcare coverage goal render the concept more central to public health (Sobel et al., 2016; World Health Organization, World Bank Group, and OECD, 2018). The importance of quality has sparked major changes in healthcare organizations. Recently, these developments have led to an aspiration to reorient services towards the patient, which is known as patient centeredness (Scholl et al., 2014).

This trend can be regarded in two ways. Firstly, the reorientation has been accompanied by a desire to understand the quality of care in a comprehensive way: considering quality beyond strictly physical outcome indicators (Ayanian et al., 2016).^1^ The last two decades have witnessed the development of structural and process indicators linked to patient-centeredness. Moreover, central instruments have emerged in pay-for-performance programs (Salisbury, 2009; Stanowski et al., 2015). In the hospital context, this approach has led to the emergence of the central notion of “patient experience,” which has become a priority for healthcare leaders (Wolf et al., 2014). This concept aims to capture the whole patient’s experience during hospitalization and reduce existing differences between what is experienced by means of common indicators. Secondly, patient centeredness development has been accompanied by a diversification of the expertise used to assess services. The patient role, which was regularly considered to be passive, is becoming more active for the patients with specific knowledge. Several areas of healthcare systems need to exploit this emerging role (Pomey et al., 2015).

Patients are expected to be more involved in their healthcare at the therapeutic level. Numerous models, such as patient engagement and patient-centered care, have been proposed in an attempt to theorize this new role (Michel et al., 2020; Ortiz Halabi et al., 2020). Patients are also recognized as having a legitimate voice in the development of public policy and health research (Martin, 2009). This “new” patient engagement is expected to play a role in the organization of healthcare establishments such as patient safety (Longtin et al., 2010). Accordingly, the place given to patients in quality assessment has undergone a major change. Patients are currently expected to play an active role in the deployed quality procedures (Bombard et al., 2018; Pomey et al., 2009). The literature is therefore largely concerned with the plurality of the mobilized knowledge and the approach in which it is jointly mobilized (Pols, 2014).

The emerging emphasis on the patient’s view of the hospital and its organization has given rise to a wide range of initiatives. The widespread use of satisfaction surveys or questionnaires, such as Patient Reported Experiences Measures, is part of this movement, but there are doubts regarding the way in which professionals are using them (Boyer et al., 2006). Moreover, the numerical representation of the patient voice keeps individuals at a distance. Other types of initiatives physically involve patients. Research on this subject has revealed a certain diversity of practices such as involvement in quality or hospital management committees, in working groups on short-term projects, or in different types of advisory panels (Liang et al., 2018).

A significant number of studies has regarded patient and public involvement schemes from the viewpoint of the factors that encourage them or the performance they achieve (Bombard et al., 2018). The studies often deplore the difficulties of implementation or the lack of evidence as to the actual improvement in the resulting quality. This study aimed at assessing these systems from the viewpoint of their functions and operation rather than their performance and optimization. As a starting point, we investigated the case of patient and family advisory councils^2^ (hereafter referred to as PFACs) in general care hospitals in the French-speaking area in Belgium (Voz et al., 2021). We begin by presenting our research problem and our methodology. We will then present the results of our analysis of how PFACs work and what they tell us about the functions performed by these arrangements. We will show that an organization’s openness to a mechanism such as a PFAC can be understood as contributing to the fulfilment of its immune function. We will show how this function is operationalized by the deployment of a second-order observation system, made possible by the choice of a “decision premise,” and how this increases the organization’s irritation capacity for a specific system-environment relationship (i.e., the organization system-user environment pair).

## 2 Problematization

### 2.1 Quality: from improvement to absorbing uncertainty

In the classic sense, quality procedures aim to improve a service or product. From this viewpoint, the understanding of quality is naturally positive with no possible reservations. Viewing quality solely in terms of its dedication to a certain performance level immediately raises the concern of the “vision of the best” that is generally adopted. In this sense, quality procedures represent “hybrid forums” in which different types of stakeholder with diverse expectations and interests confront each other (Jobert, 1992; Callon et al., 2011). Various case studies have demonstrated this viewpoint, analyzed the way in which groups position themselves in relation to quality, and ensured that they have control over its definition (Robelet, 2001). Distinct, even antagonistic, normative visions co-exist in hospitals; they are, among other things, dependent on institutional logics linked to the profession, market, state, and company in relation to the self-positioning of the players (Martin et al., 2021). Depending on the individual, the participation processes are justified in different ways (Martin, 2008). From the stakeholders’ viewpoint, the objectives pursued through quality procedures, as well as their evaluation, will therefore always depend on this logic and their interests. Restricting the analysis of quality procedures to the question of their performance forces us to situate ourselves at the level of the participating actors starting from the performance of the procedures which prevents the analysis from going beyond a certain number of normative starting points and understanding the actual contribution of the systems. However, hospital teams have a genuine need to understand the systems they are experimenting with (Bergerum et al., 2020).

### 2.2 Quality as a contingency formula

The quality concept is highly flexible (Cochoy, 2001). The quality-embracing organizations are confronted with the void left by what is a meta-standard rather than a standard with a content of its own. Quality is not the function of the hospital system, a performance, or even a symbol, but rather a pragmatic principle that serves to self-observe and self-describe the system. The idea of quality can be regarded as a “contingency formula” (Luhmann, 2019).

To embrace the notion of quality, hospitals must deploy various designed procedures to define the meaning of quality in terms of external accreditation, internal audits, writing intervention protocols, calculating and monitoring indicators, and submitting questionnaires to beneficiaries (such as PREMs, PROMs, and satisfaction surveys). All these procedures presuppose acceptance of the quality standard, without predicting the resulting decisions and whose interests will be favored; the idea of quality has been “canonized” within the hospital system.^3^ Thus, it is not regarded as a program (a selection criterion), but rather a validity claim. Quality necessarily implies evaluation; thus, the organization must be able to observe how different users assess the quality of the offered services. From the point of view of the social systems theory, this observation raise a question: How can a hospital organization blindly operate within an inaccessible environment?

Three key points of the luhmannian systems’ theory are worth recalling here.^4^ Firstly, the luhmannian systems’ theory move away from a distinction between totality and parts towards a distinction between system and environment. Secondly, social systems are operationally closed. They can only refer to elements of the system. Thirdly, social systems have boundaries. They have a dual function of linking with and differentiating from the environment. Luhmann’s general theory of social systems has made a major contribution to the theory of organizations (Chernilo, 2002; Luhmann, 2006). This brings us to the previous question. Given these theoretical points, the operational closure of social systems, and in this case of a hospital organization, excludes any possibility of direct observation of its environment.

"(…) it could be advisable to replace the unreserved endorsement of innovation (in a positive sense) by the recommendation, in view of the uncertainty absorption that is operating anyway, to maintain and cultivate the irritability of the organization" (Luhmann, 2018).

The question stems from a general challenge facing any complex system: the problem of absorbing uncertainty. Following on from Luhmann,^5^ this question leads us to rethink quality as being linked to the absorption of uncertainty, and no longer as a challenge linked to improvement or innovation (Meyer et al., 2013). The challenge of uncertainty arises in every social system, and therefore in every organization, because of the knowledge/ignorance pairing (Luhmann, 2018). Owing to its operational closure, the organization cannot directly realize its environment. However, it must be able to act within this unfathomable, indecipherable environment.

### 2.3 The PFAC as the hospital’s immune system

The accomplishment of the immune function, which is deployed by one or more of the organization’s sub-systems, enables the hospital system to operate despite the lack of knowledge regarding its environment. The immune function for the hospital system is the capacity for the system to treat external irritation as information for future decision. It is based on the enhancement of the hospital system’s sensitivity (its irritability) to its environment. External criticism, i.e., the irritations, of the hospital is a communicative event that disrupts or tests the organizational system. The reception of these irritations as information, such as irritations that can make a difference (induce change) within the system, is essential to its maintenance. For these external criticisms to be dealt with by the organization, the organization must be able to transform these irritations into information. Absorbing uncertainty implies increasing the organizational system’s capacity for self-irritation, an essential characteristic of an immune system. Opening an organization to a mechanism such as a PFAC can be viewed as contributing to the organization’s immune function, which is by no means metaphorical.

The function is first performed by sorting between self and non-self, an operation common to all recognition systems.^6^ The hospital environment is in fact made up of several other distinct systems. Thus, the system (organization)/environment (hereafter S/E) differentiation is essential. The procedure, as a communicative process generating differences (Luhmann, 2001) conveys a distinction and an indication as to which side of the difference the system considers legitimate (Seidl and Becker, 2006). The procedure renders it possible to distinguish between the different social systems that make up the hospital (such as the therapeutic interaction, professional, and organizational systems), as well as indicating the identity of those concerned by the procedure. The PFACs act as a decision premise:^7^ observing the difference between a particular system (the hospital organization) and determining its relevant environment.

Thus, performing the immune function requires overcoming the impossibility for the organization of directly observing the difference between itself and its environment. One of the characteristics of an immune system is precisely that it manages without any knowledge of the environment. It only registers internal conflicts, where it deals with irritations in the system by continuous self-observation, where it continuously observes the S/E difference within the system. Access to this observation requires the implementation of a second-order observation that involves organizing the observation of the observation of which the system is the object.

To achieve this, the sub-system dedicated to immunization must deal with the re-entry challenge (Luhmann, 2021). To be able to observe the S/E difference, the organization should re-introduce this difference within itself by activating the potential for contradictory communication (between a system and its environment) to produce insecurity (or irritation) by the PFAC and makes the hospital system tolerant to structural insecurities.

Finally, all these operations enable the immune system to function by forming automatic responses (or “antibodies”) that are case-specific and therefore more “sensitive” (self-adapted) to the environment. The hospital constantly communicates its organizational identity, such as brochures and innovative projects, which are all self-descriptions that simplify its complexity and unify or homogenize its diversity (Luhmann, 2018). Self-descriptions must be perceived as “problem-free” by those outside the organizational system. The dissemination of self-descriptions offers it its own identity.

## 3 Materials and methods

### 3.1 Methods

Quality initiatives involving some form of patient and public involvement can be observed in various countries in different manners. Among the most involving initiatives are schemes where groups of patients are installed as members and regularly invited to sit on the board for several years. In the United States, many hospitals have set up PFACs (Herrin et al., 2016). In France, hospitals have been legally obliged to set up user committees since 2012 (Pomey and Ghadi, 2009). In Belgium, PFACs emerged “spontaneously” in 2015 at the initiative of certain hospitals, without any legal framework requiring or supervising the practice. Since then, this type of assembly has spread throughout Belgium. Three case studies (Yin, 2009) of PFACs have been conducted in the French-speaking region in Belgium.

### 3.2 Description of cases

The three monitored PFACs are based in three general care hospitals in the French-speaking region in Belgium. Two PCs were set up in 2015, while the last one was set up later in 2021. The first PFAC is based in a public hospital with a “university character” and is made up of seven patient members and two professional members. It is organized by an employee in the “Strategy, Quality and Strategic Project Monitoring Department.” The second PFAC is in a public university hospital. It is theoretically made up of 15 patient members and four professional members. According to the hospital’s organization chart, it has a direct link with the organization’s medical management. The third PFAC has been operating in a private non-university hospital since 2022. It is coordinated by two professionals from a sub-section of the “Continuous Quality and Safety Improvement Department”; six patients are members in that PFAC, along with the quality director, three professionals from this department, and a patient association representative (see Table 1).

**Table 1:** Characteristics of the three studied cases.

	Case	1	2	3
Hospital characteristics	Type	General care	General care	General care
	Sector	Public	Public	Private
	Academic affiliation	Academic character	Academic	Non-academic
	Number of beds	868	1,098	903
PFACs characteristics	Year of creation	2015	2015	2022
	Hospital affiliation	Strategy management	Medical directorate	Quality department
	Number of members (patient-professionals)	9 (7–2)	19 (15–4)	10 (5–5)
	Meeting frequencies	Quarterly	Monthly	Almost Monthly

### 3.3 Data collection

The fieldwork took place over 3 years between 2019 and 2024. The communication process at work was observed directly in two ways. Firstly, most PC meetings were observed between December 2018 and November 2023.^8^ Notes were taken of all the observation sessions, including the present members, discussed subjects, content of the debates, and certain exchanges. Secondly, a large amount of documentation was collected to compile the articles of association, minutes of meetings,^9^ activity reports and various written documents such as articles, communication brochures, and opinions. The communication process at work was then indirectly observed through semi-structured interviews.^10^ The first two cases were the subject of two sets of interviews. The first series of interviews were exclusively conducted with members of the PFACs, both patients and professionals. These initial interviews dealt with the interviewee’s pathway to a hospital and then to a PFAC, what the interviewee had to do there (both in the hospital and PFAC), and his or her expectations of the PFAC’s place in the hospital. In the second stage, the members were re-interviewed and people peripheral to the PFACs, such as management, medical board, and professionals who had had occasional contact with a PFAC, were added to the sample. The reason for undertaking further interviews was to identify the latent functions of the PFACs and deepen our understanding of the roles taken on by the participants. The third case appeared later and could therefore only be the subject of a series of interviews, with members as well as people peripheral to the PFAC. This case is confirmatory in nature, in relation to the first two simultaneously investigated cases.

### 3.4 Data analysis and work processes

Data analysis was carried out throughout the data collection process. The aim was to capture the common functioning of systems such as the PFACs, despite the different hospital contexts in which they operate. Two main phases marked the analysis of the material.

The beginnings of analysis were marked by abductive reasoning (Peirce, 1992). Our initial exploration of the material attempted to provide the best possible explanatory hypothesis for the observed situations by following the question: “How do PFACs work?” Open coding was used to develop the initial hypotheses (Strauss and Corbin, 2018). Intermediate analytical summaries were used to discuss the most promising interpretative avenues as well as the unexplored areas of material and the limits of the material which is still being collected. The end of this stage was marked by the emergence of the hypothesis of an irritation function fulfilled by PFACs.

Then, the analysis was deepened in a more deductive way to overcome the fallibility of the hypotheses being abductively put forward (Lipscomb, 2012). The second phase of analysis focused on deepening the idea of irritation, both by reading Luhmann’s texts and continuing to analyze the empirical material. Gradually, through a process of axial coding (Strauss and Corbin, 2018), the phenomenon of irritation became that of immunization, and its main categories were the differentiation between social systems, second-order observation, representation of users in the organization, and multiplication of the organization’s antibodies.

While the two phases of analysis are separately presented in the current study for the purposes of writing, the process involved many successive phases of reading, analysis, and fieldwork, where iteration is continuously at the heart of the research approach.

## 4 Results: immunizing the hospital

Initially, we analyzed the S/E difference indicated by the procedure. Then, we assessed how this difference is observed within the system. Finally, in the last section, we evaluated the way in which the system provides itself with responses to future irritations, and we highlighted how the PFAC subsystem enables the hospital system to become immune to one of its environments.

### 4.1 Differentiation

Different social systems make up the “hospital” (in the common sense of the term) and constitute it as a hybrid plural social entity such as the system of therapeutic interaction, the professional system, and the organizational system (Freidson, 1970). PFACs have to manage this plurality. Whenever plurality is experienced as unity by members and users, the PFAC’s procedure can only suffer from a reference to a single system-environment pairing. The communicative process at work in the PFACs, through the differentiation of roles, is responsible for indicating the relevant difference on which to work. While the deployment of PFACs does not therefore lead to any future decisions, it does lead to a premise of decisions that the operational functioning of the PFAC must take into account (Luhmann, 2018). At the same time, this premise reduces observation to this angle and accepts the increased complexity of this new observation angle of the specific relationship between the hospital organization and its environment of users.

#### 4.1.1 Situating the relevant environment by differentiating roles

All PFAC members, whether patients or professionals, play a variety of social roles depending on the context in which they operate. The procedure requires the exclusion of the pre-existing roles, while its operation requires role specificity where the roles of the PFACs are differentiated from the surrounding roles. These roles are differentiated through various formal and informal processes specific to the PFACs. As evident by our observations and interviews, the internal procedures of the PFACs fulfil a vital function of differentiation; when a member points out that it is not a question of being a member of a patient association or when another member is excluded because he is too involved with a group of patients with a certain pathology. The different roles within the PFACs are continuously identified and dealt with through communication. The yielded outcome conveys the S/E difference that is needed to be observed by the procedure.

"We had already paid a lot of attention to this in recruitment, in relation to emotions and the illness experienced… because at the beginning [of the PFAC], some people really mixed everything up and were a bit like in therapy…". Interview extract, professional member, PFAC 2.

The most formalized procedures explicitly reveal themselves as dividers of social roles. For example, with respect to the recruitment process, which is essential to avoid “mixing everything up” as one of our interviewees put it, each PFAC has its own recruiting methodology. However, in all the methodological approaches, a preliminary interview takes place between at least one PFAC representative and the candidate member. During these interviews, a widely shared selection criterion is the “constructive” nature of the hired person. Despite its ambiguity, this criterion acts as an upstream filter to sort out who is suitable for, and what can be discuss in the PFACs. Recruiters frequently hear stories of people who are not selected owing to their very long individual care pathway or their conflictual relationship with the hospital. From a chronological point of view, this screening method is the first and most exclusionary filter in a member’s career. However, this sorting is repeated in other ways throughout the life of the PFACs, as explained in some of the examples below. It is crucial for the PFAC sub-systems that the relevant S/E difference is referred to within the PFAC and that its participants can temporarily acquire other roles in other circumstances. After identifying the dividing function, we will address what these procedures entail.

##### 4.1.1.1 In search of a consensual position: de-politicizing criticism

The changing patient identity in the 21st century led to the possible politicization of healthcare and recognition of a collective patient identity.^11^ Building on these foundations, patient activism has developed and become a force to be reckoned with in the political system.^12^ PFACs could be regarded in this light with regards to politically representing patients against one or more groups with divergent interests such as physicians, careers in general, and hospital managers.

Association activists gravitate around the PFACs, where some members are members of an association and a PFAC (their latter membership is not dependent on the former one) and one of the monitored PFACs has a seat for a patient association representative. During one of this PFAC’s meetings, the representative spoke in a militant tone, referring to their “interest in creating common positions” and her desire to “structure the PFAC” (observation of PFAC 2 meeting, 15 January 2019). Two elements characterize the role of activist in our observations and interviews. The first is the idea of identifying positions “to be defended,” which situates the relationship with the hospital or its professionals in a form of power struggle, and the second is holding “common positions,” which presupposes the existence of a collective on whose behalf to express oneself. A somewhat confrontational exchange during a PFAC meeting provoked a reaction that rather articulates the place of this role on the PFAC:

"The debate livened up among the members. The coordinator points out that it's good that the PFAC is being challenged, but that within the PFAC there are divergent opinions, "just like in society"." Observation notes, 21 May 2019, PFAC 2.

The coordinator’s speech was very brief at the time and might seem anecdotal. However, she underlined an essential argument that social diversity must be represented in the PFAC. This excludes any possibility of deploying a militant role in representing a collective. The PFAC procedure is not a part of the health policy system. Although the PFAC’s opinion sometimes takes a unified form in a written document, it does not exceed being a compilation of individual comments. Moreover, moments of collective production, such as writing an article on behalf of the PFAC or organizing a symposium, are moments conducive to tension. The inestimable diversity of experiences remains an inescapable fact from which the supposed system richness and sometimes its regrettable weakness are derived.

The tension present in interviews between the idea of representing a collective and that of representing oneself spreads to all the members and the periphery of the PFACs. The problem is neither fully addressed nor resolved by the members. It is not uncommon for members to say “we represent the patients of this hospital,” and for other members, whether patients or professionals, to strongly reject this statement. The ambiguity is particularly illustrated by the regular reference to the image of trade unions in companies. For some, the trade union model is an example to follow, while for many others, it is a matter of avoiding this model at all costs. The fears that are regularly expressed by professionals at the launch of the PFACs suggest that they are apprehensive about the demands PFACs might make, the conflictual nature of their actions vis-à-vis healthcare professionals, or the “patients’ union” role the PFAC might play in the organization. Regardless of the members’ opinions on the subject, the role of activist, or to a lesser extent representative, is made inaccessible to them by the procedure itself.

PFAC members do not base their presence on democratic legitimacy; they do not carry the voice of a community of patients. Recognition of the unity of this group is not accessible to the hospital system whose environment is characterized by an elusive complexity. Accordingly, it cannot recognize any organized, unified form. Two other social systems of reference appear in our material, which are those of the therapeutic relationship and hospital organization.

##### 4.1.1.2 Care and the self: de-personalizing criticism

Every social space is structured around differentiation of roles that can take the form of a pair of complementary roles: such as the priest and believer in church and the pupil and teacher in school. Similarly, the patient-caregiver role pair is evoked at the hospitals. The duo constitutes part of the specific social system of the therapeutic relationship (Parsons, 1952). All the patient members in the PFAC have had prior experience of several therapeutic relationships in one or more hospitals. However, as the previous interview extract indicated, it is not the therapeutic relationship that is referred to in the PFACs. During the meetings, the regular interventions calling on people not to talk about “their case,” to “stand back,” or not to pour out their emotions, mark a separation between everything that gives content to the role of patient and what is expected of PFAC members.

A particularly illustrative episode supports the hypothesis that any communication (irritation) emitted in the PFAC must go through a de-personalization process. One of the PFACs had a dedicated space each month in the hospital journal, where it wrote a few paragraphs on the issue’s theme. For an issue of the magazine dealing with the emergency department, a patient member opted to write about a very negative experience in the department. After heated exchanges between the communications department, the professionals on the PFAC, and patient members, the text was finally abandoned and replaced by a more consensual text. During this episode, some spoke of censorship, while others questioned the form of the text. From an analytical point of view, the case revealed how the procedure sets aside certain constituent features of the patient’s role. The episode was concluded with a note in the minutes of a work meeting stating that:

"It's important that the PFAC is able to express itself on subjects that are close to its heart and that the soul of the PFAC shines through in its writing. So there's no question of being dictated to in terms of style or content, although it's understood that you'll also have to adapt to the communication channel run by the hospital. Writing an article in the hospital newspaper is still a great showcase for the PFAC, and it would be a shame not to do so." Extract from Minutes 20 April 2021, working meeting, PFAC 2.

The fact that the minutes of the meeting concluded in this way suggests that the procedure ignores two features of the patient’s role. Firstly, the balance between the purpose of the PFAC and the subjective experiences of its members (*the heart and the soul*) is at the center of the problem of communication for the hospital, as it is for the members of the PFAC—who may tend to confuse their problem with that of the PFAC. The subjectivity of situations experienced by the individuals, in this case the patients, cannot be tolerated in the PFAC. Secondly, the use of the showcase image is the underlying reason for all the tension surrounding this critical episode. Whereas similar statements in a meeting may only result in reframing, the public nature of the affair catalyzed the tensions that such a role-playing error can generate. Being part of a private relationship is the second characteristic of the patient’s role. In this case, however, an event that was considered to be private was made public; we are “washing our dirty laundry in a press article,” as the head of the PFAC put it. That’s not what PFACs are for; they should not come in with an “egg to peel,” as another professional member put it. The therapeutic relationship remains a private one, which should not be publicized within a body such as the PFAC. All the observed PFACs ask their members to respect the anonymity of the reported situations and the confidentiality of exchanges.^13^

##### 4.1.1.3 The organization’s user: indication of the expected role

The positions taken from the roles discussed above are authorized in PFACs, but are entirely filtered. As long as criticism is voiced internally—the importance of internal criticism will be discussed below—role confusion is easily channeled.^14^ Consequently, criticism of the work or the care relationship is a regular feature of discussions. These criticisms are, however, channeled and referred to other mechanisms of treatment in the environment of the PFACs.^15^ By excluding the social roles of patients or activists, and referring them to the environment of the procedure, the PFACs indicate the role that needs to be assumed: that of user of a health service. The term has become widespread over the last few decades, replacing the term administered in the relationship between individuals and public services (Bizeau, 1997; Chevallier, 1985). The transition between the two terms reflects a change in this relationship: the individual is no longer just a beneficiary, but is involved in appropriating the services.

"The quality nurse visiting the PFAC today said: "Sometimes we're very supra. (…) And then, as you are experts". She has come to collect post-it notes as part of a nationwide quality campaign: "What's important to you is important to us" Observation notes, meeting 12 June 2019, PFAC 1.

The fact that a campaign dedicated to quality focuses on “what’s important to you” highlights the turning point marked by the figure of the user. It is no longer enough to think up a “good” service, divorced from any real-life experience; the service must be perceived as quality by each of the beneficiaries who became users. Thus, appropriation by individuals is central, and the singularity of the service experience is central to the figure of the user. The procedure poses a choice between singularity and community. Patients share a common experience and have been represented for several decades by patient associations. They anchor the fact of being a patient in a well-defined relationship with several significant others, including the various healthcare professionals, and in the history of that relationship. The relationship with the organization as a user has no collective history. As a head nurse and PFAC member told us, the relationship is about:

"Everything that [the user] experiences, from the moment they make their appointment to the cleanliness of their room, their meals, contact with the social worker, the timetable for their return, the reception, so it's not limited to contact with the nurse, with the doctor" Interview extract, coordinator, PFAC 1.

This is a very common statement made by everyone who is directly or indirectly involved in the PFACs. Beyond the statements, the indifference of the services whose experience is to be evaluated can be observed in the wide variety of tasks carried out by the PFACs such as rereading a pre-hospitalization brochure, testing waiting room equipment, making recommendations to the registration department, or exchanging views with the head of the dietetics department. What matters is usage regardless of the provided service. The user figure is therefore indifferent to the specificity of one or other aspect of the hospital. Through their discussions, the PFACs focus their work on the experience of all the services deployed by the hospital to enable its users to connect to it. Rather than focusing on the specifics of a particular service relationship, such as a therapeutic relationship, the procedure involves the members in the totality of what the hospital is.

By opting for singularity and comprehensiveness, the PFAC procedure establishes the user as the reference role. This is the relevant environment, whose difference from the system must be observed. The other side of the difference, the system, can be observed through the self-descriptions of which it is both the subject and object.

#### 4.1.2 System identity and self-description

As previously highlighted in this study, the system to which the patients’ PFAC procedure refers to is neither that of the therapeutic relationship, nor what we have called the political system of health. The requirement to represent the user role implies that the procedure can be understood as referring to a very specific social system: that of the hospital organization. As an organization, the hospital is involved in producing self-descriptions designed to present itself as a unit. Several self-descriptions of the same social system can co-exist: the hospital will describe itself as an efficient organization for its subsidizing powers or as an attractive working environment for its future and current healthcare professionals. In the specific relationship between this system and its environment of users, however, a particular identity prevails.

"For more than 30 years, our hospital has cared for patients and their families by promoting partnership and close collaboration with them" Extract from the PFAC's presentation brochure, PFAC 1.

Vis-à-vis its environment of users, the organization spreads the idealized image of an organization with a human face, i.e., the humanization of services. When a patient member of a PFAC is given the floor at a symposium, when the PFAC has a dedicated page in the hospital newspaper, or if the hospital publicizes the launch of its PFAC, the hospital organization is communicating its identity as an organization that listens to its users and is close to them. In PFACs, the self-description of a humanized organization serves as the main identity of the represented organization, and it is based on this partial representation of the hospital as an organization with a “human face” that the organization can be observed as in a PFAC. Accepting this self-description of the organization is fundamental to the possibility of observation. At no point is this self-description expected to be called into question. The process involves the observation of the organization’s environment of the various images of the hospital “as if” it was an organization with a human face. In this sense, the PFAC procedure is an important producer of identity, as we shall explore in more detail in the final section of the results.

### 4.2 Handling the problem of ignorance by switching to second-order observation

Although the S/E difference (between the organization and its users environment) is clearly identified by the PFACs procedures, the direct observation by the organization is nonetheless difficult. On daily basis, the patients are confronted with the hospital’s communications, its posters, brochures, letters, and forms. These communications generate numerous misunderstandings, criticisms, and questions. The organization has no direct access to observing its own differences. Professionals on the PFAC share this challenge by saying things like “we are a bit too focused on our work,” or “we are professionals, we do not have your patient viewpoint” (observation extracts, PFAC 1). Irritations from the environment do not automatically become information that can be processed by the system: therein lies the problem of ignorance for any organization, and the resulting uncertainty. To deal with this, the organization must overcome the impossibility of first-order observation.

#### 4.2.1 Observing the S/E difference: self-observation and self-irritation

As our analysis suggests, the PFACs function as self-observation tools for the hospital. The PFACs organize this through situations such as the one described in the observation note below.

"Today's meeting was devoted to the presentation of a new center dedicated to the well-being of oncology patients, "La Grande Cabane". A nurse, a psychologist and two clinical beauticians were present to present it, in addition to the professionals usually present. A discussion ensued with the PFAC, without any specific requests being made. All comments were well received. Observation notes, meeting of 11 September 2019, PFAC 1.

The usual work situation described in the excerpt from the aforementioned observation illustrates the main procedure of the PFACs: observing what the hospital produces in terms of its user environment. As one quality manager put it: “We had all the brochures, and we came up with the idea of putting them through the PFAC. We did not say ‘Here are the brochures, tell us what you think’. We told them what to look for in the brochures… there was a little training and a checklist, saying ‘Here’s what we expect from you’“(Interview extract, quality manager, PFAC 3). First you must get people to observe what you want, the way you want them to. Then, you must give yourself the opportunity to observe how users observe the hospital which is the fundamental principle of how PFACs work. As one head of administration pointed out in an interview:

"The idea is to present it to them…". Does it upset you too? Is it important to you or not? So, every idea is really tested with the PFAC." Interview extract, patient services manager, Periphery, PFAC 1.

Accordingly, the observer role is essential to the system. However, this role is not envisaged or provided for in the documents governing the procedure. Formally, the PFACs are made up of only two groups, patient and hospital representatives—we will present this representation in the next section. Without observation, however, the procedure is rendered as meaningless.^16^ The PFAC procedure is presented as a privileged place for observation: a place from which we look, as well as a place that we look at. Without self-observation, there can be no self-irritation, the procedure’s second objective:

"It allows us to say to ourselves "be careful, we're reminded of this in the PFAC, in the surveys sometimes it comes across too" so let's be vigilant about this because patients are asking for this. So, yes, it sometimes allows us to redo the attention points. In the routine of our work, in the speed at which we sometimes must move forward and work on our files, we can stop at a given moment and say to ourselves 'There's this and that which comes out of the PFAC, the investigations, the mediation or complaints department'…". Interview extract, coordinator, PFAC 1.

The coordinator’s description of the benefits of the PFAC (among other tools used by the organization) clearly shows how it helps to increase the organization’s capacity for irritation. The PFAC, like the other mentioned tools, makes the organization sensitive to its environment. Moreover, maintaining a boundary with its environment renders this as possible. The PFACs grant certain professionals the opportunity to closely observe the difference between the hospital and its environment to increase the hospital’s sensitivity to its surroundings: it enables it to identify “points of attention” whenever necessary. The “work routine” and “speed” with which professionals operate blind them to their environment—the organization of work has no place in its day-to-day operation for its environment. To alleviate this challenge, the procedure seeks to bring the difference between the inside and outside environments to life as often as possible, so that the hospital organization can observe it and become aware of it.

#### 4.2.2 The need for dual representation

Self-observation can only work by internally reproducing the difference between the hospital system and its environment. To make a difference in the hospital—i.e. to bring about change within the system itself—a dual representation is required: that of the environment and the system.

"Today, a symposium is being prepared in which the PFAC is taking part. A patient asked a doctor on the PFAC: "Can't you represent us? "I'm from the hospital, not you". Later, the patient asked again: "Can you replace me on the stage?", and the doctor replied: "I don't think that would go down very well". Observation notes, meeting of 11 December 2018, PFAC 2.

As presented in our material, self-observation presupposes that the difference between two distinct poles (patient and professional) is represented. The exchange between a patient member and a professional member might seem surprising at first sight as they are both members of the PFAC. This gives rise to the question as to why would it be problematic, or even unpleasant, for one to replace the other? Being a PFAC member must not obscure the fundamental difference (between inside and outside), otherwise the system would lose the intended purpose. It’s not just a question of representing one of the poles of the difference, but rather the difference itself. In this respect, and contrary to what the players may say, the PFAC is not a body for expressing the “voice of patients.” More precisely, the PFACs are places where the difference between the users and hospital is represented. Carrying out this quality procedure involves interpreting two roles specific to the PFAC: representing the hospital and representing the hospital environment.

"We created the "Greeting" project, which is a project to welcome people with special needs. (…) The project was already well developed, and we were going to do it, but I think it's still important to get their feedback. And it's a first public test. How will the public react to this project (…). I use them as testers, as guinea pigs, and they help me" Interview extract, Patient Services Manager, PFAC 1.

Representing the hospital is achieved through the presentation to the PFAC of a representation that is always partial: a project that it has, a brochure that it deploys, equipment that it makes available, or a campaign that it prepares. As in the case of “La Grande Cabane” in the first observation extract, visiting professionals take it upon themselves to materialize the hospital during meetings. Through the *Greeting* project mentioned by the interviewee, a partial materialization of the hospital is mobilized in a PFAC. What is at the center of the exchange is never the hospital as a whole, but its partial and situated materializations. The representation of the hospital must be matched by the representation of its environment, through the intermediary of “the guinea pigs, the testers.” This is the second role to be interpreted, and the members who embody this role must be able to react like “ordinary mortals,” as one interviewee put it. In this sense, to represent the hospital environment, it is much more a question of participating as someone else rather than on behalf of others. The nurse manager’s use of the term guinea pig highlights the fact that the difference is made through representation in a similar manner to that in a laboratory. While representation of the hospital does not pose a huge problem (its concretization through projects or presentation materials emanating directly from the organization itself and therefore following its own procedures), representation of the environment confronts the PFACs with a paradoxical situation.

### 4.3 Representing users in the organization: dealing with the practical problem of re-entry

The PFAC procedure requires the hospital organization’s environment to be represented. But how can we represent an environment that is by nature unstructured? The user environment is *a priori* impossible to represent. The singularity of each user experience, characterized, as we have observed, by the individual appropriation of undifferentiated services, is irreducible to any form of structuring. This is a sensitive point for a number of people, both members and outside the PFACs, who question the “poor representativeness” of the members. At a symposium on PFACs, a hospital director took the floor to share his experience of the PFAC in his hospital, and came up with a question to which he gave no answer: “Do they [the members] really represent anything?” (Notes from observations, PFAC symposium, 14 December 2018). In an interview, the same director doubtfully told us: “Here, in the institution, we have 1,100 doctors and 1,800 nurses, and we are going to set up a small PFAC with 10 people, 6 of whom will be present” (interview extract, medical director, PFAC 1).

Of course, part of the problem stems from the depoliticization of the aforementioned criticism. Nonetheless, the system has to face up to this concrete problem: how can it represent its own environment? In other words, how do you deal with the practical problem of re-entry? An analysis of how the PFACs work shows that overcoming this paradox means that each PFAC member must become a member of the hospital organization itself. It is on this last condition that the hospital environment can legitimately express itself within the hospital itself, or rather that the S/E difference can be validly represented within the system.

#### 4.3.1 Become a member and socialize with the organization

Including the PFAC in the hospital’s organization chart, systematically sending meeting minutes to management, holding meetings on the hospital’s premises, and presenting the PFAC on the hospital’s website are all signs that the PFAC belongs to the organization. The roles of the procedure depend on this.

"I don't think it was clear either: "user", "patient", "patient representative". It's not clear to everyone. So, in the new internal regulations and in the fact that we're now signing a contract with the hospital, there's a legal link with specific missions, which will make it possible to reframe things a bit more" Interview extract, professional member, PFAC 2.

The director’s comments followed a conflict during which the role of the PFAC, the recognition of its members, and the legitimacy of their contributions were widely questioned. The solution that was concluded to put an end to the discussions was to further entrench the link between the members and the hospital by signing a proper contract. Such a contract would reinforce the essential fact: to express themselves validly, people involved must be members of the organization. The blurring of the lines between the user, patient, and patient representative mentioned at the beginning of this extract highlights the need for differentiation, which is met by the indication assumed by such a contract. Although none of the studied PFACs drew up such a contract, a number of internal procedures within the PFACs contributed to the differentiation of roles that is necessary for the PFACs to function: formalized recruitment procedures, signing of charters, or internal rules.^17^ However, these preliminary moments are not enough to become and retain the member status. The life of the PFACs is marked by a process of socialization into the hospital organization.

"An exchange between the coordinator and a patient member began about her participation in the PFAC. The patient said that it would take some time for her to accept that her voice was legitimate: "At the beginning, I would have been more observant, I would have suffered. Now I feel legitimate. (…) Participation in the group legitimizes my presence on other [hospital] PFACs. (…) You need time to get your bearings, to understand what's expected of you". Observation notes, 19 January 2023, PFAC 3.

The time mentioned by the member is not sufficient alone to conduct the work; “understanding what is expected of us” is achieved through a process of gradual socialization into the hospital organization. It even enables them to feel “legitimate on other hospital PFACs.” This process takes several forms. Firstly, training programs for each of the members (patients) are an essential part of their integration. As one interviewee put it: “They need to be familiar with the hospital world so that they can propose things that are acceptable or feasible” (interview extract, quality and safety coordinator, member, PFAC 3). The training programs vary from one hospital to another. They always include at least one session on confidentiality and anonymity. Depending on the case, group dynamics, patients’ rights, or participation in various conferences, enrich members’ training. Secondly, the many explanatory and reframing interventions by the most experienced members for the most recent members also contribute to socialization, by explaining to them who to contact and how, or reminding them that “the hospital is very hierarchical.” Establishing a direct link with the organization, as well as the socialization process, ensures, as we discuss below, that the representation of the environment operates internally, within the organization.

#### 4.3.2 Respecting the boundaries of the organization: representing the S/E difference internally

This process enables everyone, patients and professionals alike, to become socialized into the organization at its most formal setting; thus, they become anchored in a particular system, that of the hospital organization. Differentiating oneself from extra-procedural roles to become a member of the PFAC sets people on the path to belonging to the organization itself.

"The aim is above all to enable patients to express themselves about what's going well and what's going less well (…) But yes, there are bound to be rules, because we're in a hospital institution (…). As for us hospital professionals, there are bound to be rules. The rules also include confidentiality, which is an important rule. Anything discussed within the PFAC cannot be released, especially if we're talking about people's names, if we're talking about departments. It seems logical that this should not go beyond the walls of the hospital (…). I think they understand that not everything is possible. You can't extend the walls of the hospital" Interview extract, PFAC coordinator, PFAC 1.

Our observations and the aforementioned interview extract in particular, highlight the importance of providing a specific framework for self-observation of the difference. It must be carried out internally, within the confines of the organization. This gives rise to the importance of the socialization process at work within the PFACs. As far as the PFAC respects the contours of the organization and organizes itself within it, i.e., by taking account of its own procedures, the difference between the organization and its environment can be freely expressed and observed. The fact that the coordinator mentions that we sometimes talk about the names of people or departments (when this is not normally expected) does not pose a problem as long as “it does not go beyond the walls of the hospital.” She stressed the importance of this condition for the PFAC’s operation. The members must “hear” the organization in order to express themselves. The system must ensure that they remain within the “walls” of the hospital and that they respect the system boundaries. Although the image is also used in this context in a very concrete way by the coordinator to imply that we do not have the place we want, the image reveals the importance of being situated within the organization itself to represent difference.

### 4.4 Multiplying automatic responses to external aggression

The functioning of the self-observation and self-irritation processes described above fulfils a particular function for the organization. More than the traditionally stated function of improvement, we argue that it contributes to the organization’s immunization to one of its environments, in this case that of its users. More specifically, this involves increasing self-knowledge and preparing responses to non-self. The deployment of the PFACs is not a precursor to any decision: the implementation of the PFACs does not entail any details regarding the “antibodies” that will be proposed. On the contrary, it opens up the possibility of new, as yet undetermined decisions.

#### 4.4.1 Fueling the content of the notion of humanity: a contribution to self-knowledge

As presented earlier, the representation of the hospital in PFAC involves the presentation of some of its materializations. These represent acts of communication of the organization’s self-descriptions. In the case of PFACs, the hospital describes itself as an organization with a human face. However, the notion of humanization is just as empty as the notion of quality. Giving it content is the first function performed by the PFACs; this is a real work on identity (on the organization’s self).

"[On re-reading a brochure on the side effects of radiotherapy] As a medical oncology patient in remission, may I offer my personal opinion on the subject? Cancer treatments are heavy, aggressive and sometimes even violent. So patients need a little gentleness, comfort and hope. Your brochure should be like a companion, providing that gentleness, comfort and hope. So I also think that this imperative tone is inappropriate" Extract from advice submitted, PFAC 1.

The opinion issued following a request for the brochure to be re-read by the PFAC deplored the lack of “gentleness, comfort and hope” in contrast to the harshness, aggressiveness, and violence of the treatments. The opinion expressed urges the humanized organization to recognize the violence of the treatments, and to show itself to be gentle and comforting. This would be the attitude of a humanized organization. The diversity of tasks undertaken by PFACs, and of what they produce, are, *a priori*, difficult to reduce to a single common denominator (beyond the stated general objective of improving quality) such as proofreading brochures, testing equipment, speaking at public events, and taking part in various other committees. All the opinions issued contribute to the organizational development, where all these opinions contribute to building a specific representation of the “human quality” of the hospital organization’s services. Through our observations, representations are shared about hospitals, professionals, and patients. The hospital must be able to go beyond its biophysical jurisdiction and direct its services towards the patient (as a unique human being), rather than towards the disease. Furthermore, professionals are expected to listen and take into account the patient’s view point. As for the patient, who was traditionally represented as a passive being who is subject to medical authority, he or she is currently expected to behave as an “active social patient,”^18^ responsible, informs himself, learns for himself, and engages in dialogue with healthcare professionals. The transversal vision formed by these three idealized forms of the patient, professional, and hospital, lies in a strong expectation of adaptation.

"I'm not sure we have the same definition of quality of care on either side. So most of the questions [from the PFAC] revolve around comfort, respect for the individual, psychological and social well-being. I don't think that's what doctors see as the quality of care. (…) These are different representations, at every level. Patients can't understand that we have economic concerns. That's what we hear first. I experience this every day. The patient must leave! Her justified length of stay has been exceeded" Interview extract, professional member, PFAC 2.

Feeding such a humanized self-description of the hospital may seem obvious to the user environment. However, feeding this image locally, within the organization itself, is necessary to enable it to distinguish what it is - or should be - for one of its particular environments. Other self-descriptions circulate about it: about its profitability, as mentioned in the extract, or about its great expertise. Discussions in PFAC help to differentiate between these self-descriptions.

#### 4.4.2 Developing a repertoire of antibodies

The entire process described above must *ultimately* fulfil an essential function for the organization: preparing a repertoire of responses to the irritations of its environment. PFACs must provide the organization with the antibodies that enable the humanized hospital to withstand criticism from its users. For example, in the second PFAC, the advice given after a brochure had been proofread urged the organization to adapt its writing because “the imperative tone is inappropriate,” in view of the fact that “Cancer treatments are aggressive and sometimes even violent. Patients therefore need a little gentleness, comfort, and hope” (Extract from the opinion of PFAC 2). The opinion commits the hospital to changing one of its representations to enable it to maintain its identity as a humanized organization. If we “pretend” that the hospital has a human face, the “imperative tone is inappropriate.” By taking account of the way it is observed by its environment, the organization acquires the means to adapt to maintain one of the identities conveyed by its self-descriptions.

"And that's why it's interesting that there's a hospital mediation service (…). And it's interesting that there's this PFAC and that there's the 'patient satisfaction' group, because all these complaints, all these demands, if they weren't considered, could lead to conflict. But here, it's framed" Interview extract, Patient Services Manager, Periphery, PFAC 2.

The representation of difference may follow different procedures. Many of our interviews draw parallels between what professionals observe at mediation or in patient satisfaction surveys and the content of exchanges in the PFAC. The importance of these procedures is that the observed difference is taken into account, as the head of a patient service indicated in the interview. Being able to “manage” (organize the procedure for) the occurrence of “complaints and demands” (irritations) provides the organization with the means to avoid “conflict.” The dissatisfaction relayed internally by a member about the imposed reduction in the use of a service (see above) enables the organization to anticipate the response to be made to protect itself from conflict. The function remains the same when a coordinator talks about organizing a “welcome week” for professionals. The story of the many criticisms levelled at the way people are welcomed at the hospital leads to the need to raise awareness among workers and prevent potential conflicts.

"I mean, it's good to have users' opinions, otherwise how can we improve? We couldn't improve (…) I like reading the complaints too. Because it teaches us special things. (…) We have to keep improving. We'll never be 'perfect'. There will always be things … and the world evolves, medicine evolves too, contacts evolve (…) You can't sit in your office and think to yourself, 'Well, maybe this is good for the patient'" Interview extract, hospital general management, PFAC 2.

The interviewed director uses the lexicon of improvement. However, she focused on the learning and awareness-raising she had gained from the PFACs. The image of the person alone in his office reflects the difficulty created by the operational closure of the organization; without opening up to its environment (making itself sensitive to it), the organization cannot protect itself from its irritations. The operation of PFACs helps to maintain a boundary with its environment, and to be able to manage its complexity, the changing “world, medicine, contacts.” Additionally, it alerts its workers (healthcare professionals or hospital managers) or its objects (brochures, chairs, or signage) to the difference it maintains with its environment so that they are ready to react.

## 5 Discussion

We aimed at deepening our understanding of how quality and patient and public involvement systems work within healthcare organizations. We also aimed at contributing to the “empirical opening” of Luhmann’s sociological theory (Helge Becker and Seidl, 2007). Using the case of PFACs in general care hospitals in Belgium, our analysis describes the immune function fulfilled by these participatory mechanisms within the hospital organization. Several operating principles underpin this essential function: differentiation between social systems, the shift to second-order observation, the re-entry of the S/E difference, and the multiplication of the organization’s antibodies. From this point of view, PFACs appear to fulfil a crucial function for organizations, well beyond the simple discussion of the “nuts and bolts” of the organization’s structure (Glickman et al., 2007). This analysis obviously does not exhaust the issues raised by the deployment of PFACs. Future analyses could focus on the changes these mechanisms have had on power relations within hospitals. Still others might look at the impact of the institutionalization of these PFACs on patients’ lay knowledge. Nevertheless, understanding these mechanisms as the realization of the immune function of an organization allow us to discuss implications for the notions of patient and public involvement and quality in organizations. We propose to structure them around the three key points of Luhmanian theory mentioned in the introduction: differentiation, boundary and operational closure.

To begin with, there are several lessons to be learned from the centrality of the differentiation mechanism. The understanding of the differentiation mechanism at work in the deployment of PFACs initiatives can help to clarify the future arrangements deployed. As we mentioned in the introduction, there are several schemes that can lay claim to patient and public involvement, such as patient association or patient expert. Their scope is often unclear. Questioning empirically the specific social system with which they communicate would undoubtedly help to clarify the functions performed by each. Moreover, the centrality of the differentiation operation in the procedure to which individuals are taking part enlighten the ambivalence or hybridity of professional identities within the sector, and the tensions they may generate at the individual level. Numerous articles have examined the identity work carried out by professionals in the face of upheaval in the healthcare sector (Mcgivern et al., 2015; Martin et al., 2021). These articles tell us how professionals and users negotiate the transition to a so-called hybrid identity, which involves professionals in a form of liminality (Beech, 2011). In a PFAC, this hybridity remains a particularly difficult matter, both for patients and some professionals. This certainly partially explains why patient and public involvement can be a “precarious task” (Hasselbladh and Bejerot, 2007). Our own findings reveal that, while participation in this type of scheme helps in creating new social roles, the operation of the scheme itself maintains the boundaries between the different roles that members are required to play. Within a given scheme, the way it operates determines a particular role, by differentiating it from others, and over and above individual motivations for taking part. When participating in a given procedure, at a given time, nobody can “be hybrid.” Accordingly, taking on a role in this type of system implies a certain level of reflexivity and an ability to play different roles (Mead, 1934). The present analysis questions the calls for “comprehensiveness” that abound in public health. Working on quality, rather than encompassing “everything” that goes on in a hospital in an undifferentiated way, the PFACs draw a line between the distinct objects of hospital organization, therapeutic relationship, and relations of power and knowledge between professional and nonprofessional groups.

Beyond these implications at the individual level, the systems theory approach and the thesis of the immune function at work in the PFACs contribute to shed light on our understanding of the quality deployment in organizational systems.

To continue, the place occupied by borders in the analysis gives us food for thought about the openness of organizations. The participatory component of these quality mechanisms could seem as an opening of hospitals. Indeed, the case of the PFACs provides food for thought on *open organizing*, defined as “a dynamic organizing principle along the primary dimension of transparency/opacity and the secondary dimensions of inclusion/exclusion and distributed/concentrated decision rights” (Splitter et al., 2023). PFACs could be considered as an advance in terms of transparency, inclusion, and distribution of decision rights. Moreover, they are fairly typical illustrations *of unmanaged* openness (Whittington and Yakis-Douglas, 2020). Although the PFACs are organized by the hospitals themselves, there are few constraints either external to the organization or within it, which define their boundaries. Implementation is performed by those who volunteer or are appointed to “open up” the hospital to the “patient voice.” In this situation, our results highlight the fact that “opening up” the organization requires the establishment of strong boundaries (of which the PFACs are an illustration). The case bears a stimulating resemblance to the observation of the need “to bound openness” in the case of online forum moderation (Lingo, 2023); as we have seen, the re-entry of the S/E difference become possible only because of a strong socialization to the organization. In the light of our results, however, it seems that the relationship between openness and closure should not be regarded as antagonistic but complementary. In this sense, participation mechanisms for quality fulfil both a separating and a linking function, two functions that are specific to the boundaries of social systems (Luhmann, 2010). The operational closure of an organization, by maintaining boundaries, is the essential condition for its openness to the environment.

Finally, the operational closure of the organization and the impossibility of direct access to the organization’s environment presented in the results also contribute to thinking about the way in which change is conceived. PFACs are cases of systemic change management, as opposed to conventional change management (Lies, 2020). The latter envisages the success of change management through a series of direct interventions dependent on planned decisions taken by individuals. Our results suggest that the PFACs proceed differently: by making themselves sensitive, and more tolerant, to irritations in their environment, the PFACs help to maintain a particular type of self-description of the organization, that of a humanized organization. By positioning PFACs on the periphery of the hospital organization, they help in immunizing the hospital organization against its patient environment (i.e., to criticism from outside). Rather than thinking of change from a conventional perspective as dependent on either a top-down or bottom-up dynamic (By, 2005) thinking about change in a systemic way provokes us to question its processes in the relationship between the center and periphery of an organization. The way in which peripheral sub-systems are linked to the organizational center, such as through *loose* coupling (Beekun and Glick, 2001), becomes a crucial issue in the success (and understanding) of change.

## Data Availability

The original contributions presented in the study are included in the article/supplementary material, further inquiries can be directed to the corresponding author.

## Footnotes

^1^Adopt a global view of care, in which the medical-technical act is only one stage among others, as in the case of health-promoting hospitals (WHO, 1991).

^2^We decide to use here the designation PFAC despite the local use of ‘Patient Committee’ to cope with the use of the international literature.

^3^As the idea of justice within the legal system (Luhmann, 2019).

^4^For an overview of Luhmann’s theory, see (Orianne, 2019).

^5^The luhmannian approach is opposed to the theory of action in which the ideas of individuals (their intentions and objectives) are the main units of understanding (Luhmann, 2010, 2018). Whereas other approaches propose to perceive the organization in terms of what is stable or structural, in a form of essentialism, Luhmann views the organization as intrinsically subject to ruptures and discontinuities. In this manner, it is what enables it to remain stable that can be questioned.

^6^Read The Remembered Present: A biology of Consciousness (Edelman, 1989).

^7^Luhmann defines a decision premise as following: “By “premise” we mean a precondition that can no longer be checked when used; or rather that, although its relevance for the problem at hand plays a role, its truth does not” (Luhmann, 2018).

^8^In the first PFAC: Five meetings observed from November 2018 to October 2019, sixth meeting observed post-covid19 resumption in March 2022. Second PFAC: Eleven meetings observed from December 2018 to October 2019, a twelfth observation was made when the PFAC stopped in June 2022. Third PFAC: Four meetings observed from January 2023 to January 2024.

^9^Including meetings at which observation was not possible.

^10^First case: 6 interviews from December 2020 to April 2021, 6 interviews from March to June 2022. Second case: 8 interviews from July 2020 to March 2021, 5 interviews from March to June 2022. Third case: 6 interviews from March to September 2022.

^11^See, for example, N. Dodier, Leçons politiques de l’épidémie de sida (Dodier, 2003).

^12^The term is understood here in the broad sense already used by Dodier (2003), of the field of power relationships between constituted groups.

^13^The public nature of interventions also raises a second problem, which we will address below, that of re-entry.

^14^For example, resistance to depersonalisation can occur, as when a patient member recounts that a doctor calls his patients by their names in the corridor from his office, and that a professional member insists: “Tell me who it is, so I can go and check it out” (meeting observation, 22 November 2018, PFAC 1). The role confusion that arises within the PFACs can have various outcomes and be dealt with in different ways.

^15^Such as hospital mediation for requests concerning patients’ rights, or directly to healthcare professionals for criticisms specific to the therapeutic relationship.

^16^The importance of observation in the PFACs is a criterion that strongly differentiates our cases, as is the hospitals’ satisfaction with the success of their respective PFACs. In PFAC number 1, no one really took on the role of observer, leading to the realisation that the PFAC served no purpose - which led to its closure, 6 years after it was launched.

^17^The struggles in the second PFAC (to obtain a hospital badge, car park access cards and certain forms of compensation) seem to be symptomatic of the need for participants to be recognised as *de facto* members of the organisation in order to have a legitimate voice. The fact that these struggles are emerging in one of the PFACs where the legitimacy of the opinions expressed is regularly called into question is revealing.

^18^As in other areas of public intervention: (see Orianne and Beuker, 2019).
